# The impact of VKORC1-1639 G>A polymorphism on the maintenance dose of oral anticoagulants for thromboembolic prophylaxis in North India: A pilot study

**DOI:** 10.4103/0971-6866.80360

**Published:** 2011-05

**Authors:** S. S. Rathore, S. K. Agarwal, S. Pande, T. Mittal, B. Mittal

**Affiliations:** Departments of Genetics and CVTS; 1 Sanjay Gandhi Post Graduate Institute of Medical Sciences, Lucknow, Uttar Pradesh, India

**Keywords:** Allele frequency, drug dose, genotype, polymorphism, VKORC1

## Abstract

**BACKGROUND:**

The dose requirements for oral anticoagulants in thromboembolic events are influenced by promoter polymorphism in the VKORC1 gene. However, limited data are available on the influence of the polymorphism in various Indian populations. The present study aimed at determining the relationship between the VKORC1-1639 G>A genotypes and maintenance doses of oral anticoagulants for therapeutically stable INR values in patients taking Acitrom after valve replacement surgery.

**MATERIALS AND METHODS:**

Fifty patients from the northern Indian region were genotyped for VKORC1-1639 G>A by polymerase chain reaction and restriction fragment length polymorphism. Means of the weight-normalized daily Acitrom dose were calculated for every patient.

**RESULTS AND DISCUSSION:**

The VKORC1 1639G>A minor allele frequency in the study population (*n* = 50) was found to be 22%. The patients with a wild type genotype required the maximum drug dose as suggested for full functionality of the enzyme. Heterozygous patients were found to have an intermediate drug dose and the patients with a variant homozygous genotype had the minimum maintenance drug dose requirement. These findings are in concurrence with the effect of the promoter polymorphism on vitamin K epoxide reductase activity.1639G>A minor allele frequency in the study population (n = 50) was found to be 22%. The patients with a wild type genotype required the maximum drug dose as suggested for full functionality of the enzyme. Heterozygous patients were found to have an intermediate drug dose and the patients with a variant homozygous genotype had the minimum maintenance drug dose requirement. These findings are in concurrence with the effect of the promoter polymorphism on vitamin K epoxide reductase activity.

**CONCLUSION:**

The VKORC1-1639 G>A status can be indicative of establishing the therapeutic dose of oral anticoagulants in Indian patients.

## Introduction

The use of oral anticoagulants has become one of the most common therapies to treat patients suffering from thromboembolic disorders. Warfarin/Acitrom and related coumarins are widely used in thromboembolic prophylaxis. The subtherapeutic and supratherapeutic use of these drugs is associated with an increased risk of clot formation and bleeding, respectively. This is because of the narrow therapeutic index of these anticoagulants. There is much concern to ensure improvement in the safety of oral anticoagulant therapy, and many efforts have been made for this purpose. However, still, the risks for clotting and bleeding subsequent to oral anticoagulant therapy remain significantly high.[1,2] Warfarin and Acitrom are two widespread oral anticoagulant drugs that are used to maintain the International Normalized Ratio (INR) within the therapeutic range. However, the dose requirements of these anticoagulants vary between individuals as well as within an individual, depending on the dietary intake or as a result of variations in pharmacokinetics and pharmacodynamics, compliance, etc.[3] In recent years, common genetic variations in vitamin K epoxide reductase (VKOR) have been discovered to significantly influence the oral anticoagulant maintenance dose requirements. Acitrom (or Warfarin) inhibits vitamin K epoxide reductase,[4] specifically the VKORC1 subunit,[5,6] thereby diminishing the available vitamin K and vitamin K hydroquinone in the tissues. Interindividual variations in the VKORC1-1639 G>A gene polymorphism has been linked to differences in the dose requirements for oral anticoagulants, A recent study compared the weight-normalized oral anticoagulant dose requirements for Chinese and Malays with that for Indians,[7] and reported the oral anticoagulant dose requirement in Indians to be higher than that in the Chinese. In this study, most Indians belonged to South India. The Indian population comprises multiple ethnic groups, and there is a need to obtain data from other regions. Therefore, we carried out a pilot study in the North Indian population to find out the relationship between the VKORC1-1639 G>A genotypes and maintenance doses of oral anticoagulants for therapeutically stable INR values in patients taking Acitrom after valve replacement surgery.

## Materials and Methods

### 

#### Patients and DNA extraction

The present study was carried out on the patients who had undergone heart valve replacement surgery and were being followed-up for oral-anticoagulant therapy. The recruitment of 50 patients (receiving maintenance oral-anticoagulant therapy with a stable, therapeutic INR between 2 and 3.5 for at least 3 months) was done from the outpatient department of Department of Cardiovascular and Thoracic Surgery, Sanjay Gandhi Post Graduate Institute of Medical Sciences, Lucknow, India, between March 2010 and August 2010. Patients aged <18 years or having diabetes, liver disease, malabsorption or chronic diarrheal conditions were excluded from the study. Patients who were receiving any of the following medications, which could potentially interact with warfarin or Acitrom, were also excluded: antiepileptics, including phenytoin and carbamazepine; antituberculosis medications, including rifampin (INN, rifampicin) and isoniazid; antibiotics, including quinolones, tetracyclines, erythromycin, cephalosporins, metronidazole and cotrimoxazole; antifungals, including fluconazole and itraconazole; lipid-lowering agents, including clofibrate and cholestyramine (INN, colestiramine); antiarrhythmics, including quinidine and amiodarone; histamine-2 blockers, including cimetidine and ranitidine; nonsteroidal anti-inflammatory agents; allopurinol; cyclosporine (INN, ciclosporin); barbiturates; and oral contraceptives.[8] In the course of warfarin/acitrom therapy, all patients were given dietary advice to avoid foods that may interfere with drug pharmacokinetics. Clinical data, including age, gender, ethnicity, body weight, medical indication for drug use and average maintenance dose, were recorded. The average maintenance dose was the mean dose during the period when two consecutive stable INR values were documented. The north Indian ethnicity was based on place of residence in the last three generations, food habits and the mother tongue (Hindi or related languages). Blood samples were collected in ethylenediaminetetraacetic acid and genomic DNA was extracted from peripheral blood leukocyte pellets using the standard salting-out method.[9] The quality and quantity of DNA was checked by gel electrophoresis and spectrophotometry using the NanoDrop Analyzer (ND-1000) spectrophotometer (NanoDrop Technologies, Wilmington, DE, USA). The ratio of absorbance at 260 and 280 nm of DNA were between 1.7 and 1.9. The isolated DNA was stored at –70°C.

#### Genotyping of VKORC1 (-1639G>A, rs9923231) allele by the polymerase chain reaction (PCR)-restriction fragment length polymorphism (RFLP) method

Genotyping of VKORC1 (-1639G>A) was performed as described by Sconce *et al*.[10] Sequences for the forward and reverse primers were 5’-GCCAGCAGGAGAGGGAAATA-3’ and 5‘-AGTTTGGACTACAGGTGCCT-3’, respectively. The PCR conditions consisted of 25 cycles of 1 min at each of the following: 94°C, 59°C and 72°C. The 5’-untranslated region (UTR) PCR product (10 μL) was digested with two units of restriction enzyme Msp1 (Fermentas, Glen Burnie, MA, USA) in a final volume of 30 μL in the appropriate digestion buffer at 37°C for at least 16 h. The digested products were visualized on 10% polyacrylamide gels stained with ethidium bromide.

#### Statistical analysis

The 95% confidence intervals were calculated using the Confidence Interval Analysis software version 1.0. The level of statistical significance was set at *P* <0.05. All the analyses were performed according to the Statistical Package for Social Science (SPSS 15.0 for Windows; SPSS Science, Chicago, IL, USA). The means of weight-normalized daily drug dose and INR were calculated.

## Results

### 

#### Allele and genotype frequencies of VKORC1 alleles in north Indian patients

The VKORC1 - 1639G>A minor allele frequency in the study population (n = 50) was found to be 22% (95% CI: 15.00– 31.07) [Table 1] . Of 50 patients, six patients were homozygous for VKORC1 (-1639 AA) (12%, 95% CI: 5.62–23.80) and 10 patients were heterozygous, having both the alleles (-1639GA) (20%, 95% CI: 11.24–33.04)

**Table 1:** VKORC1-1639 G>A genotype and allele frequencies in patients on oral-anticoagulant therapy

Genotypes	Number of subjects		Frequency	95% confidence interval
	Males	Females		
GG	22	12	68	54.19–79.24
GA	9	1	20	11.24–33.04
AA	4	2	12	5.62–23.80
Alleles	Number of alleles		Frequency	95% confidence interval
G	78		78	68.93–85.00
A	22		22	15.00–31.07

The total number of subjects genotyped for the VKORC1-1639 G>A allele is 50 (males = 35 and females = 15); total no. of alleles = 100

#### Patient characteristics, oral anticoagulation and VKORC1 gene locus

Of the 50 patients, 34 had the VKORC1-1639 GG genotype. This group of patients had six, twenty three and five cases of aortic, mitral and double valve replacement surgery, respectively [Table 2] . The mean weight-normalized daily drug dose for this group was the highest (0.069 ± 0.029 mg/kg) as compared with the patients with VKORC1-1639 genotypes GA and AA. There were 10 patients with a VKORC1-1639 GA hetrozygous genotype, with a mean weight-normalized daily drug dose requirement of 0.041 ± 0.018 mg/kg. This patient group had four cases of aortic valve replacement and six cases of mitral valve replacement. The third group of six patients homozygous for the VKORC1-1639 AA genotype consisted of two, three and one cases of aortic, mitral and double valve replacement, respectively. These patients required the least mean weight-normalized daily drug dose of 0.022 ± 0.010 mg/kg only to maintain the INR in the therapeutic range.

**Table 2:** Relationship between VKORC1-1639G>A polymorphism with maintenance dose of Acitrom

VKORC1-1639G>A	Indication, A/M/D (number)	Acitrom mean daily dose, mg/kg body weight (SD)	body weight (SD) Mean INR (SD)
GG	6/23/5 = 34	0.069 (0.029)	2.9 (0.4)
GA	4/6/0 = 10	0.041 (0.018)	2.7 (0.3)
AA	2/3/1 = 6	0.022 (0.010)	2.4 (0.4)

A/M/D indicates the number of patients who had undergone surgery for aortic/ mitral/double valve replacement, respectively. SD = standard deviation

## Discussion

The VKORC1-1639G>A minor allele frequency in the study patient group was 22%, which was significantly different from the East Asians as well as the Caucasian populations.[11] The role of genetics in oral anticoagulant dose requirement, in patients with thromboembolic disorders, has been investigated in many previous studies.[12] The daily dose requirement is the highest in the African population, while the white population required intermediate doses and the Asian population required the lowest doses. Lee *et al*.[7] reported that the mean weight-normalized warfarin dose was lower for the Chinese and the Malays than for the Indians.

At present, only clinical factors (such as age, race, body surface area, vitamin K intake, co-prescribed drugs and co-existing diseases), which contributed to 12–20% of dose variability, can be used for estimating the dose of oral anticoagulants. In the post genomic era, common genetic variations in gene VKORC1 have been discovered to significantly influence the oral anticoagulant maintenance dose requirements. Our results show clearly the impact of VKORC1-1639 G>A polymorphism on drug dose requirement to maintain a therapeutic range of INR in patients on oral-anticoagulant therapy. The patients with variant AA genotype had the least VKORC1 enzyme functionality so that there was a low availability of vitamin K and vitamin K hydroquinone in the tissues, resulting in subsequent reduced efficacy in blood clotting. This results in a decrease in the mean daily drug requirement for anticoagulation. In case of patients with the heterozygous GA genotype, the VKORC1 enzyme shows an intermediate activity and therefore a higher maintenance mean daily drug dose requirement. The highest demand of oral anticoagulant has been observed in patients with the wild type GG genotype, where the enzyme is fully functional. In conclusion, we report the frequency of the important genetic variant VKORC1-1639 G>A in a north Indian patient group that is different from other populations. We also report that the maintenance dose of the oral anticoagulant is dependent on the VKORC1-1639 G>A genotype of the individuals. Therefore, the genotype should be considered before initiating oral-anticoagulant therapies. The present pilot study was carried out in a relatively smaller number of patients and there is a definitive need to replicate it in a larger sample size as well as in various regions and ethnic groups of India before introduction in routine clinical practice.
